# *Staphylococcus aureus* CC30 Lineage and Absence of *sed*,*j*,*r*-Harboring Plasmid Predict Embolism in Infective Endocarditis

**DOI:** 10.3389/fcimb.2018.00187

**Published:** 2018-06-08

**Authors:** Jean-Philippe Rasigade, Amélie Leclère, François Alla, Adrien Tessier, Michèle Bes, Catherine Lechiche, Véronique Vernet-Garnier, Cédric Laouénan, François Vandenesch, Catherine Leport, The AEPEI Study Group, B. Hoen

**Affiliations:** ^1^CIRI, Centre International de Recherche en Infectiologie, Inserm U1111, Université Claude Bernard Lyon 1, CNRS UMR5308, ENS de Lyon, Lyon, France; ^2^Centre National de Référence des Staphylocoques, Hospices Civils de Lyon, Lyon, France; ^3^UMR-1137, Université Paris Diderot, Sorbonne Paris Cité, Paris, France; ^4^Institut National de la Santé et de la Recherche Médicale, UMR-1137, Paris, France; ^5^CIC-1433 Epidémiologie Clinique, Institut National de la Santé et de la Recherche Médicale, Centre Hospitalier Universitaire de Nancy, Nancy, France; ^6^EA4360, Apemac, Université de Lorraine, Nancy, France; ^7^Service de Maladies Infectieuses et Tropicales Centre Hospitalier Universitaire de Nîmes Caremeau, Nîmes, France; ^8^Faculté de Médecine EA 4687 Université de Reims Champagne Ardenne, Reims, France; ^9^Laboratoire de Bactériologie, Centre Hospitalier Universitaire de Reims Robert Debré, Reims, France; ^10^Service de Biostatistiques, Hôpital Bichat, AP-HP, Paris, France; ^11^Unité de Coordination du Risque Épidémique et Biologique, AP-HP, Paris, France

**Keywords:** *S. aureus*, MRSA, infective endocarditis, stroke, CC30, enterotoxin, superantigen, plasmid

## Abstract

*Staphylococcus aureus* induces severe infective endocarditis (IE) where embolic complications are a major cause of death. Risk factors for embolism have been reported such as a younger age or larger IE vegetations, while methicillin resistance conferred by the *mecA* gene appeared as a protective factor. It is unclear, however, whether embolism is influenced by other *S. aureus* characteristics such as clonal complex (CC) or virulence pattern. We examined clinical and microbiological predictors of embolism in a prospective multicentric cohort of 98 French patients with monomicrobial *S. aureus* IE. The genomic contents of causative isolates were characterized using DNA array. To preserve statistical power, genotypic predictors were restricted to CC, secreted virulence factors and virulence regulators. Multivariate regularized logistic regression identified three independent predictors of embolism. Patients at higher risk were younger than the cohort median age of 62.5 y (adjusted odds ratio [OR] 0.14; 95% confidence interval [CI] 0.05–0.36). *S. aureus* characteristics predicting embolism were a CC30 genetic background (adjusted OR 9.734; 95% CI 1.53–192.8) and the absence of pIB485-like plasmid-borne enterotoxin-encoding genes *sed, sej*, and *ser* (*sedjr*; adjusted OR 0.07; 95% CI 0.004–0.457). CC30 *S. aureus* has been repeatedly reported to exhibit enhanced fitness in bloodstream infections, which might impact its ability to cause embolism. *sedjr*-encoded enterotoxins, whose superantigenic activity is unlikely to protect against embolism, possibly acted as a proxy to others genes of the pIB485-like plasmid found in genetically unrelated isolates from mostly embolism-free patients. *mecA* did not independently predict embolism but was strongly associated with *sedjr*. This *mecA*-*sedjr* association might have driven previous reports of a negative association of *mecA* and embolism. Collectively, our results suggest that the influence of *S. aureus* genotypic features on the risk of embolism may be stronger than previously suspected and independent of clinical risk factors.

## Introduction

Infective endocarditis (IE) is a severe disease with ~20% in-hospital mortality (Duval et al., 2012). *Staphylococcus aureus*, the major causative agent of IE (Selton-Suty et al., 2012), induces severe forms that cause patient death twice as frequently as other microorganisms (Miro et al., 2005; Thuny et al., 2005, 2007). Frequent causes of IE-related mortality include congestive heart failure, multiorgan failure or embolism which occurs in 13–51% of cases (Millaire et al., 1997; Vilacosta et al., 2002; Durante Mangoni et al., 2003; Thuny et al., 2007). IE-related embolism results from the release in bloodstream of fragments from vegetations, which are masses of thrombotic, infected tissues attached to heart valves. The known risk factors for IE-related embolism are mostly linked to patient or disease characteristics such as injection drug use (IDU) or a larger vegetation size on echocardiography (Fowler et al., 2005; Thuny et al., 2007). However, *S. aureus* IE *per se* has also been repeatedly reported as an independent risk factor (Vilacosta et al., 2002; Thuny et al., 2005; Hubert et al., 2013; Rizzi et al., 2014), suggesting that microbiological, species-specific factors might be involved in embolus development and release (Durante Mangoni et al., 2003).

Indeed, most *S. aureus* isolates secrete toxins that might influence the course of IE and the risk of embolism (Vandenesch et al., 2012). Determining whether specific virulence factors are involved in this threatening complication would help predict embolism and guide preventive strategies in IE patients. Experimental studies have pinpointed the potential role in vegetation development of several staphylococcal factors, including superantigens which have been proposed to facilitate local bacterial growth by promoting immune dysfunction and chronic inflammation (Stach et al., 2016), or cytotoxic exotoxins able to kill immune cells recruited at the site of infection (Salgado-Pabón et al., 2014; Dupieux et al., 2015). These experimental findings, however, have not been confirmed in clinical studies. No epidemiological study of an association between embolism and virulence factors has been conducted so far. Strikingly, the only *S. aureus* characteristic shown to influence embolism risk is methicillin resistance (MRSA) encoded by the *mecA* gene, which emerged as a protective factor (Thuny et al., 2005; Hsu and Lin, 2007; Hill et al., 2008). However, the negative association of *mecA* with embolism has no clear biological explanation and *mecA*-positive isolates have been involved in severe IE with embolism in several case reports (Zheng et al., 2015). This current knowledge gap is possibly linked to the difficulty of conducting large-scale cohort studies of *S. aureus* IE combining clinical and microbiological molecular data. IE is a rare disease: while large cohorts have been successfully analyzed (Fowler et al., 2005), the necessity to collect and characterize *S. aureus* isolates has likely limited the sample size of genetic association studies. Moreover, the modest statistical power achievable in such small-size cohorts would counteract the benefits of current high-resolution molecular techniques such as whole-genome sequencing. It is doubtful that even cohort sizes in the hundreds would allow to reliably detect predictors of embolism among thousands of potential genetic markers (Hong and Park, 2012), especially, since these yet-unknown predictors should be controlled for confusion with known clinical risk factors.

These observations prompted us to examine potential associations of *S. aureus* characteristics with embolism during IE using a carefully selected set of candidate genes rather than a genome-wide approach. We identified, from a previous prospective population-based IE cohort in which embolic events were well-documented (Selton-Suty et al., 2012), 98 patients with *S. aureus* IE whose causative isolate could be analyzed for their genetic background and for the presence of 26 alleles involved in virulence or virulence regulation. This approach allowed testing associations with sufficient statistical power in models controlling for clinical confounders, at the expense of waiving the discovery of unexpected markers in other parts of *S. aureus* genome.

## Materials and methods

### Patient population and collection of data

Patients with IE were identified from a 1-year prospective population-based observational study conducted in 2008 in seven French regions comprising one-third of the adult French population (Selton-Suty et al., 2012). The study was approved by the institutional review board of Besançon (Comité de Protection des Personnes). In accordance with French regulations, patients were informed of the study but they did not have to provide written consent. In this cohort, 497 patients had definite IE according to Duke-Li criteria (Li et al., 2000). One hundred and thirty-two patients (26.6%) had monomicrobial *S. aureus* IE. Patients in whom *S. aureus* had been isolated upon IE diagnosis, but whose isolate could not be recovered from the frozen strain collection were excluded (*n* = 34; 25.6%). Ninety-eight patients and *S. aureus* isolates were included in the final analysis. The clinical characteristics of the 34 excluded patients were compared to those in the final cohort to detect possible biases related to strain availability.

The study endpoint was the occurrence of at least one embolic event from the onset of IE symptoms to hospital discharge. Embolism based on clinical and/or imaging diagnosis was reported among a pre-established list of complications (Selton-Suty et al., 2012). Because the date of occurrence of embolism was not consistently reported, we did not consider its delay from IE onset. The other collected variables are summarized in Table 1.

**Table 1:** Clinical characteristics of 98 patients with Staphylococcus aureus endocarditis and their association with embolism.

	**Total population (*n*, 98) *n* (%)^a^**	**Embolism (*n*, 54) *n* (%)^a^**	**No embolism (n, 44) *n* (%)^a^**	**Odds Ratio (95% CI)**	***P*-value^b^**
**DEMOGRAPHIC CHARACTERISTICS (BASELINE)^c^**					
Age ≥ 62.5 years (median)	49 (50.0)	16 (29.6)	33 (75.0)	0.14 (0.06–0.34)	<0.001
Male sex	75 (76.5)	44 (81.5)	31 (70.5)	1.85 (0.72–4.74)	0.20
**CARDIAC UNDERLYING CONDITIONS^c^**					
Underlying HD, 3 classes					
Previously known HD without prosthetic valve	28 (28.6)	11 (20.4)	17 (38.6)	0.42 (0.17–1.06)	0.14
Prosthetic valve	14 (14.3)	9 (16.7)	5 (11.4)	1.17 (0.35–3.94)	
No previously known HD	56 (57.1)	34 (63.0)	22 (50.0)	1.00 (–)	
Previous IE	4 (4.1)	2 (3.7)	2 (4.5)	0.81 (0.11–5.98)	0.83
Intracardiac device (PM or ICD)	15 (15.3)	6 (11.1)	9 (20.5)	0.49 (0.16–1.49)	0.21
**COMORBIDITIES—AT RISK PROCEDURES—MODE OF ACQUISITION^c^**					
Charlson comorbidity index, median (IQR)	1 (0–3)	1 (0–2)	2 (1–3)	0.85 (0.70–1.01)	0.07
≥1 Comorbidity	42 (42.9)	20 (37.0)	22 (50.0)	0.59 (0.26–1.32)	0.20
Diabetes mellitus	22 (22.4)	8 (14.8)	14 (31.8)	0.37 (0.14–1.00)	0.05
Cancer	11 (11.2)	4 (7.4)	7 (15.9)	0.42 (0.12–1.55)	0.19
At risk procedures <3 months	21 (25.3)	6 (14.0)	15 (37.5)	0.27 (0.09–0.79)	0.02
Mode of acquisition of IE, 3 classes					
Community-acquired cases (without IDU)	50 (51.5)	29 (54.7)	21 (47.7)	1.00 (–)	<0.001
IDU	18 (18.6)	17 (32.1)	1 (2.3)	12.3 (1.52–99.9)	
Healthcare-associated cases, 1 is not acquired in hospital	29 (29.9)	7 (13.2)	22 (50.0)	0.23 (0.08–0.64)	
**CLINICAL AND BIOLOGICAL MANIFESTATIONS OF IE^c^**					
Fever	94 (95.9)	52 (96.3)	42 (95.5)	2.48 (0.22–28.26)	0.47
Location of IE (not exclusive)					
Aortic	35 (35.7)	21 (38.9)	14 (31.8)	1.36 (0.59–3.15)	0.47
Mitral	38 (38.8)	17 (31.5)	21 (47.7)	0.50 (0.22–1.15)	0.10
Tricuspid	23 (23.5)	19 (35.2)	4 (9.1)	5.43 (1.69–17.49)	0.005
Pacemaker	4 (4.10)	1 (1.9)	3 (6.8)	0.26 (0.03–2.57)	0.25
Unknown	7 (7.10)	4 (7.4)	3 (6.8)	1.09 (0.23–5.17)	0.91
Heart failure	31 (31.6)	16 (29.6)	15 (34.1)	0.81 (0.35–1.91)	0.64
Septic shock (before surgery)	11 (11.2)	9 (16.7)	2 (4.5)	4.20 (0.86–20.57)	0.07
CRP at admission, mg/L, median (IQR)	228 (133–316)	248 (187–346)	203 (41.2–270)	1.01 (1.00–1.01)	0.01
Creatinin serum levels ≥180 μmol/L	42 (43.8)	25 (47.2)	17 (39.5)	1.37 (0.60–3.09)	0.45
**ECHOCARDIOGRAPHY^c^**					
Vegetation	87 (88.8)	47 (87.0)	40 (90.9)	0.67 (0.18–2.46)	0.55
Initial size of the vegetation, mm, median (IQR)	14 (10–20)	16 (12–20)	10 (8–17)	1.05 (1.00–1.11)	0.07
Ordinal size of the vegetation, 5 classes					
No vegetation	11 (11.2)	7 (13.0)	4 (9.1)	1.00 (–)	0.14
<10 mm	15 (15.3)	5 (9.3)	10 (22.7)	0.29 (0.06–1.46)	
≥10 mm – <15 mm	23 (23.5)	10 (18.5)	13 (29.5)	0.44 (0.10–1.93)	
≥15 mm	38 (38.8)	26 (48.1)	12 (27.3)	1.24 (0.30–5.05)	
Unknown size	11 (11.2)	6 (11.1)	5 (11.4)	0.69 (0.12–3.78)	
**OUTCOME**					
Cardiac surgery	34 (34.7)	24 (44.4)	10 (22.7)	2.72 (1.12–6.60)	0.03
Length of hospitalization, days, median (IQR)	37.5 (24–67)	35.5 (21–71)	39 (25.5–62.5)	1.00 (0.99–1.01)	0.99
In-hospital death	42 (42.9)	21 (38.9)	21 (47.7)	0.70 (0.31–1.56)	0.38
Death at 1 year	48 (49.0)	23 (42.6)	25 (56.8)	0.56 (0.25–1.26)	0.16

*CRP, C-reactive protein; EE, embolic events; HD, heart disease; ICD, implantable cardioverter defibrillator; IDU, injection drug use; IE, infective endocarditis; MRSA, methicillin-resistant Staphylococcus aureus; MSSA, methicillin-susceptible Staphylococcus aureus; PM, pacemaker*.

a *Values are numbers (percentages) unless otherwise indicated*.

b *P-value from Wald test, logistic regression*.

c *For the following variables not detailed in this present table; the P-value was >0.10: Country of birth; region of residence; central venous access; cardiac catheter; severe regurgitation; prosthesis dehiscence; cardiac abscess*.

### *S. aureus* typing by DNA array technique

Given the small sample size (*n* = 98) and the need to limit the number of model covariates to avoid type I error inflation while preserving statistical power, the genetic analysis was purposely focused on biologically relevant genetic features selected *a priori* for their potential role in IE. A subset of non-constant alleles involved in virulence and expression regulation (*n* = 22 and 4, respectively, Supplementary Table 2), was identified from the 332 target sequences probed by the StaphyType DNA array (Alere Technologies GmbH, Jena, Germany). Targets related to species identification, molecular typing, surface-expressed proteins and resistance determinants were excluded, with the exception of the methicillin resistance-conferring *mecA* gene, considered as a control covariate. DNA extraction and hybridization were performed as described elsewhere (Tristan et al., 2012). To examine the influence of *S. aureus* genetic background on the occurrence of embolism, isolates were assigned to multilocus sequence types (STs) and clonal complexes (CCs) by comparing whole-array hybridization profiles to previously MLST-typed reference strains in a dedicated database as described elsewhere (Monecke et al., 2008).

The genotypic relatedness of isolates was visualized by means of a minimum spanning tree (MSTree) based on StaphyType hybridization profiles using the complete set of targets. Briefly, an MSTree is a connected undirected graph selected to minimize the sum of marker differences over all links between genotypes (Rasigade et al., 2017). The MSTree was computed using R software version 3.2.1 (the R Foundation for Statistical Computing, Vienna, Austria) and *igraph* package version 1.1.2 (Csardi and Nepusz, 2006), and visualized using Gephi software version 0.9.2 (Bastian et al., 2009).

### Statistical analysis

Our aim was to detect associations of *S. aureus* genetic background and virulence-related markers with embolism, taking into account clinical risk factors and preserving statistical power. Models were based on logistic regression taking embolism as the outcome. Univariate analysis was used first to examine each microbiological and clinical potential predictor individually. Predictors with a Wald *P*-value < 0.1 were considered candidates for inclusion in multivariate logistic regression analysis. To account for the substantial number of variables relative to sample size and the high degree of collinearity between microbiological variables, the logistic model was regularized using a ridge regression procedure with automatic regularization parameter optimization as described elsewhere (Cule et al., 2011; Cule and De Iorio, 2013), using R software and *ridge* package version 2.2. Predictors with logistic coefficient *t*-test *P*-value < 0.1 were considered for inclusion in the final model. The final set of predictors was determined using analysis of deviance where predictors were sequentially tested for inclusion by increasing order of their *P*-value in the ridge regression model. Predictors were discarded if their inclusion did not achieve 0.05 significance in likelihood ratio test, then a final, non-regularized logistic regression model was constructed. The effect of each predictor was reported as an adjusted odds-ratio with 95% confidence interval. The classification accuracy of the final model was assessed using C-statistic (area under receiver operating characteristics curve) with 95% confidence interval based on Delong's method (Sun and Xu, 2014) and computed using *pROC* R package.

Additionally, we verified that the exclusion of non-virulence-related alleles from regression analyses did not led to discarding important predictors of embolism. To this aim, we conducted random forest analyses on the complete microarray data combined with relevant clinical predictors identified in multivariate logistic regression. We used the Boruta feature selection algorithm (Kursa and Rudnicki, 2010), which was repeatedly demonstrated to exhibit maximal performance for selecting predictors in random forest models (Kursa, 2014; Kumar and Shaikh, 2017). In the Boruta algorithm, permuted copies of each predictor (the so-called shadow variables) are added to the dataset before estimating the random forest model. After several repetitions of this step, the distribution of the Z-score (importance measure) of each predictor is compared to the distribution of the maximal Z-scores among the shadow variables. Predictors whose Z-scores are significantly higher or lesser than the maximal shadow variable Z-score are classified as important or unimportant, respectively, while other predictors importance is left undetermined. Of 332 alleles, 118 were non-constant and included in analysis. The Boruta technique was performed with R package *Boruta* using 500 repetitions of random forests of 1,000 trees.

## Results

### Patient characteristics

The clinical characteristics of the 34 patients with *S. aureus* IE whose bacterial strain was not available did not differ from those of the 98 patients included in the final analysis (Supplementary Table 1). Patients had a median age of 62.5y, 75 of them (76.5%) were male and 42 (42.9%) had at least one comorbidity (Table 1). Embolism was diagnosed in 54 patients (55.1%), of which 31 (57.4%) had symptomatic embolism while the remaining diagnoses were based on imaging tests. Embolism was mostly cerebral (*n* = 25, 46.3%) and pulmonary (*n* = 20, 37.0%). Other locations including spleen, kidney and peripheral arteries were found in 29 patients (53.7%). Eighteen patients (33.3%) had multiple embolic events in several locations. In-hospital death occurred in 21 patients with embolism (38.9%). This mortality rate was comparable to that of patients without embolism (47.7%, *P* = 0.38).

### Clinical and bacterial predictors of embolism

In univariate analysis, the clinical factors possibly associated (*P* < 0.1) with embolism were, by increasing *P*-value, younger age (less than the 62.5 y cohort median), mode of IE acquisition including IDU, tricuspid IE location, CRP level at admission, history of a procedure at-risk of IE in the previous 3 months, diabetes, Charlson comorbidity index, septic shock and vegetation size (Table 1).

**Figure 1:**
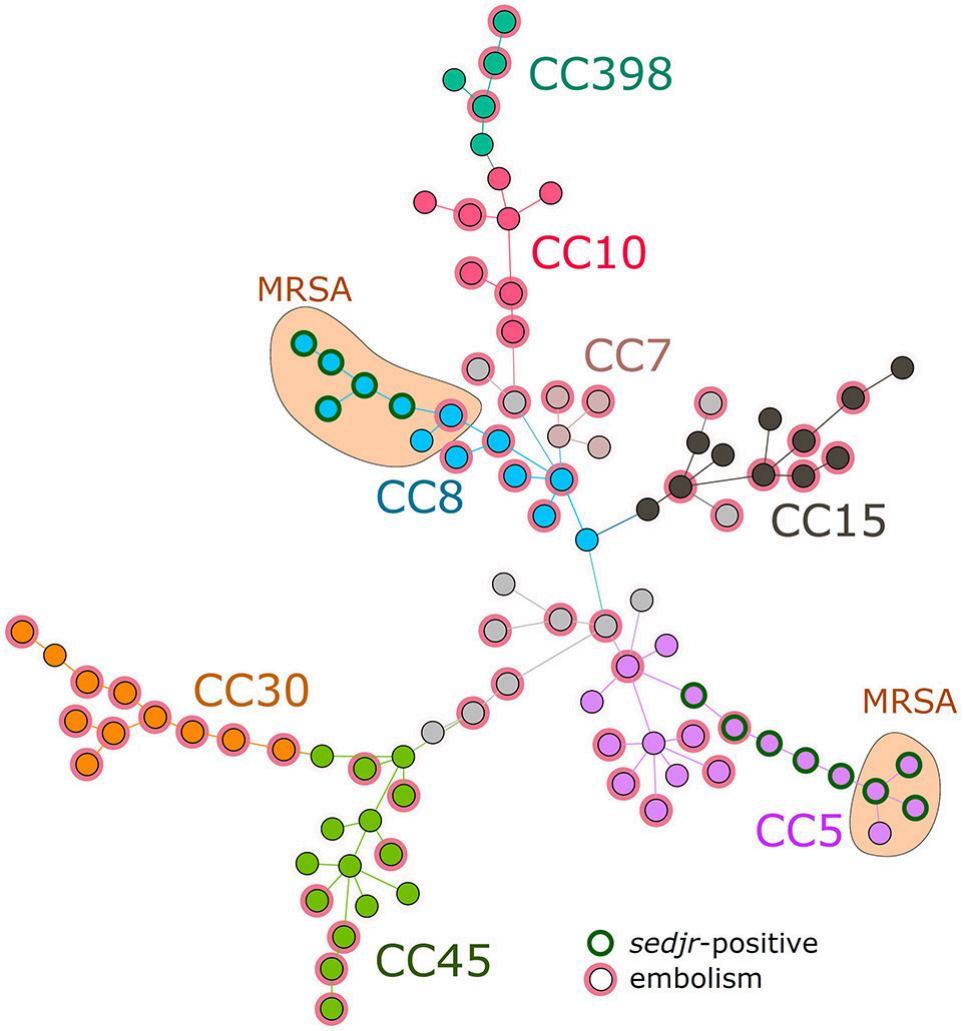
Genotypic relationships and characteristics of 98 *S. aureus* isolates from endocarditis patients with and without embolism. Shown is a minimum spanning tree where connections between isolates are selected as to minimize the total number of genotypic differences in the tree, based on DNA arrays targeting 332 genes and alleles. Colored marks are used to indicate embolism-associated isolates and those harboring *sedjr*, a set of plasmid-borne enterotoxin-coding genes negatively associated with embolism in the cohort. Gray marks denote isolates belonging to rare clonal complexes (CCs). MRSA, methicillin-resistant *S. aureus*.

*S. aureus* methicillin resistance based on *mecA* gene presence was negatively associated with embolism (Table 2), consistent with previous findings (Thuny et al., 2005; Hsu and Lin, 2007; Hill et al., 2008). Associations between genetic background and embolism were moderately significant, with an enrichment of CC30 and *agr* III (to which CC30 belongs) groups in embolism-associated isolates. Among the five virulence-related predictors with Wald *P*-value < 0.1, four were superantigens while one predictor, namely *lukE*, was a component of the LukED cytotoxic exotoxin known to provoke host cell lysis by targeting chemokine receptors (Tam et al., 2016). One of the superantigen-related genetic markers was the *tst1* gene encoding toxic shock syndrome toxin 1 (TSST-1), previously suspected to aggravate vegetation growth in a rabbit IE model (Stach et al., 2016). The three other superantigen determinants were *sed, sej*, and *ser*, which encode enterotoxins involved in food poisoning and are harbored by the same pIB485-like penicillinase plasmid (Bayles and Iandolo, 1989; Shearer et al., 2011). Because *sed, sej*, and *ser* were always harbored together in the same isolates, they were hereafter referred to as a single predictor *sedjr*. The *sedjr* markers were negatively associated with embolism, with an odds ratio of 0.05 (95% CI, 0.01–0.41; Table 2). Interestingly, *sedjr* and *mecA* were strongly associated (*P* = 1.16 × 10^−6^, Fisher's exact test) and both were only found in CC8 and CC5 isolates (Figure 1).

**Table 2:** *S. aureus* genetic characteristics in 98 endocarditis patients and their association with embolism.

				**Univariate analysis**	
	**Total population (*n* = 98)**	**Embolism (*n* = 54)**	**No embolism (*n* = 44)**	**OR (95% CI)**	***P-*value^a^**
**GENETIC MARKERS WITH WALD TEST *P*-VALUE<0.10**					
*mecA*: alternate penicillin binding protein 2, defining MRSA	11	1 (9.1)	10 (90.9)	0.06 (0.01–0.52)	0.01
*tst1:* toxic shock syndrome toxin 1	8	7 (87.5)	1 (12.5)	6.40 (0.76–54.2)	0.09
*sed*: staphylococcal enterotoxin D	13	1 (7.7)	12 (92.3)	0.05 (0.01–0.41)	<0.01
*sej*: staphylococcal enterotoxin J	13	1 (7.7)	12 (92.3)	0.05 (0.01–0.41)	<0.01
*ser*: staphylococcal enterotoxin R	13	1 (7.7)	12 (92.3)	0.05 (0.01–0.41)	<0.01
*lukE*: leukocidin E component	44	20 (45.5)	24 (54.5)	0.42 (0.17–1.00)	0.05
**GENETIC BACKGROUND**					
***agr*** **(4 groups)**					
I	50	28 (56.0)	22 (44.0)	1.08 (0.49–2.39)	0.86
II	33	14 (42.4)	19 (57.6)	0.46 (0.20–1.08)	0.07
III	14	12 (85.7)	2 (14.3)	6.00 (1.26–28.46)	0.02
IV	1	0 (0.0)	1 (100.0)	– (–)	0.99
**CLONAL COMPLEX**					
CC15	11	6 (54.5)	5 (45.5)	0.98 (0.28–3.44)	0.97
CC30	11	10 (90.9)	1 (9.1)	9.77 (1.20–79.7)	0.03
CC398	5	3 (60.0)	2 (40.0)	1.24 (0.20–7.74)	0.82
CC45	15	7 (46.7)	8 (53.3)	0.67 (0.22–2.02)	0.48
CC5	19	7 (36.8)	12 (63.2)	0.40 (0.14–1.12)	0.08
CC8	13	6 (46.2)	7 (53.8)	0.66 (0.20–2.13)	0.49
Others^b^	24	15 (62.5)	9 (37.5)	1.50 (0.58–3.84)	0.40

*MRSA, methicillin-resistant Staphylococcus aureus*.

a *P-value from Wald test, logistic regression*.

b *CCs classified as others were CC1, CC10, CC12, CC121, CC20, CC25, CC7, CC8/ST7, CC88, CC9, CC97, ST188 and ST6*.

**Figure 2:**
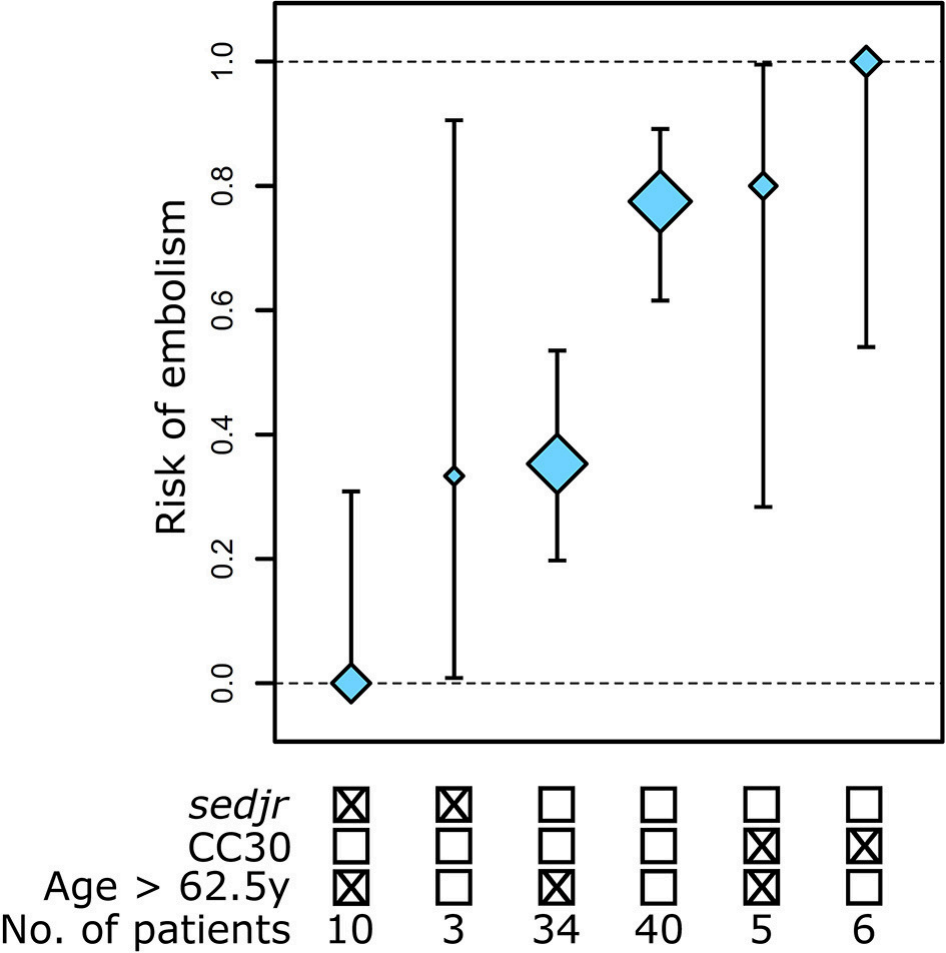
Stratification of the risk of embolism in *S. aureus* endocarditis patients based on independent clinical and microbiological predictors. Three predictors of embolism were identified using regularized logistic regression followed by analysis of deviance. Shown are the proportions of patients with embolism in each risk group with 95% binomial confidence interval. Mark size is proportional to group sample size.

To better delineate the clinical and microbiological predictors of embolism, variables with Wald *P* < 0.1 (*n* = 15) in univariate analysis were jointly analyzed using regularized regression (Table 3). In this multivariate model, the previously suspected predictor *mecA* (Hill et al., 2008) was not independently associated with embolism (*P* = 0.19). Only four factor coefficients had *P* < 0.1, namely (by increasing *P*-value order): *sedjr*, age < 62.5 y, IDU and CC30 genetic background. Upon applying analysis of deviance to these four predictors, only IDU failed to reach significance (*P* > 0.05) and was discarded (Table 4). The final, non-regularized model included *sedjr*, age < 62.5 y and CC30 as predictors, with a C-statistic of 0.81 (95% CI, 0.73–0.89). Noteworthy, the inclusion of *mecA* in this model did not bring additional information, indicating that *mecA* significance in univariate analysis was due to its association with *sedjr*. In our cohort, thus, the major independent predictors of embolism in IE patients were a younger age and a causative isolate belonging to CC30 and/or not harboring *sedjr*, while *mecA* had no measurable independent influence. The accumulation of predictive factors steadily increased the risk of embolism, from 0% in older patients with *sedjr*-positive, non-CC30 *S. aureus*, to 100% in younger patients with *sedjr*-negative, CC30 *S. aureus* (Figure 2).

**Table 3:** Clinical and microbiological predictors of embolism in patients with *S. aureus* endocarditis in a regularized logistic regression model.

	**Adjusted OR (95% CI)**	***P*-value**
**CLINICAL PREDICTORS**		
Age ≥ 62.5 y (median)	0.01 (0.00–0.57)	0.024
Intravenous drug use	104.57 (1.70–6450.58)	0.027
Tricuspid endocarditis	12.04 (0.23–623.67)	0.217
Charlson comorbidity index	2.39 (0.06–102.00)	0.649
Diabetes mellitus	1.81 (0.04–76.02)	0.755
Septic shock (before surgery)	1.62 (0.04–72.48)	0.803
CRP at admission, mg/L	0.76 (0.02–32.95)	0.885
Initial size of the vegetation, mm	1.04 (0.03–39.80)	0.983
**MICROBIOLOGICAL PREDICTORS**		
*sedjr*	0.01 (0.00–0.43)	0.018
*mecA*	0.06 (0.00–4.02)	0.193
*tst1*	3.71 (0.08–168.70)	0.501
*lukE*	2.77 (0.06–119.10)	0.595
**Agr group**		
*agr* I (reference)	–	–
*agr* II	0.20 (0.00–8.65)	0.400
*agr* III	4.37 (0.09–209.30)	0.455
*agr* IV	0.08 (0.00–4.14)	0.208
**Clonal complex**		
CC30	27.23 (0.66–1121.93)	0.082
CC5	0.61 (0.01–26.75)	0.795

*Regularization parameter Lambda = 0.026, selected automatically based on 8 principal components (Cule and De Iorio, 2013)*.

**Table 4:** Final predictive model of embolism in patients with S. aureus endocarditis.

**Predictor**	***P*^a^ (ridge regression)**	***P*^b^ (analysis of deviance)**	**Adjusted OR (95% CI)**
*sedjr*-positive *S. aureus*	0.018	<0.0001	0.073 (0.004–0.457)
Age > 62.5 y	0.024	<0.0001	0.137 (0.048–0.358)
Injection drug use	0.027	0.051	–^c^
CC30 *S. aureus*	0.082	0.015	9.734 (1.527–192.8)
*mecA*-positive *S. aureus*	0.193	0.671	–

a t-test for coefficient significance in logistic ridge regression model with 15 candidate predictors

b likelihood ratio test for model improvement in unregularized logistic regression;

c *predictors with P > 0.05 in analysis of deviance were excluded from the final model*.

**Figure 3:**
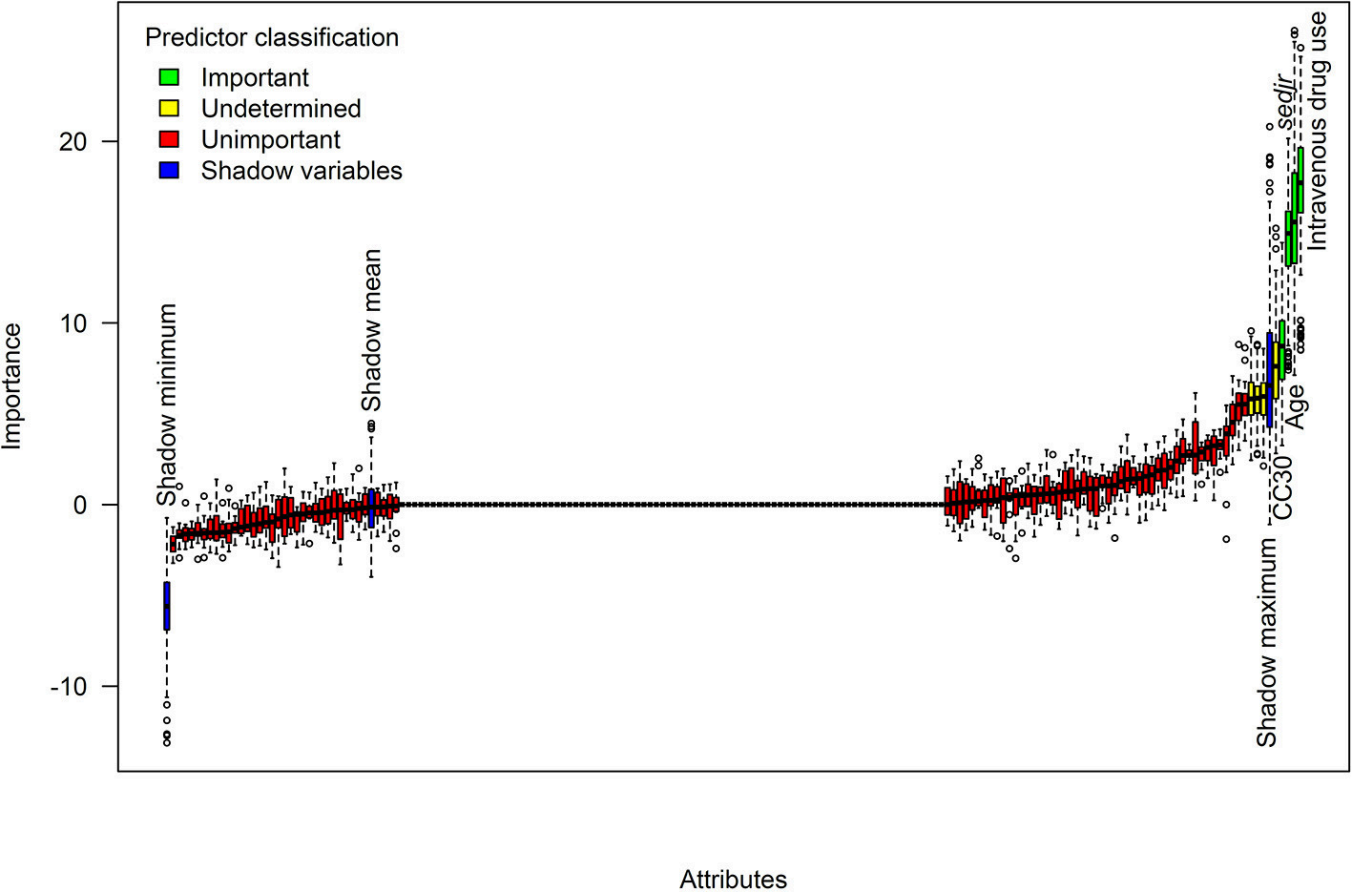
Identification of predictors of embolism using random forests. Shown are the distributions of random forest importance measures for 118 microbiological predictors and 2 clinical covariates. Importance measures were obtained from 1,000-tree random forests and Boruta feature selection algorithm with 500 repetitions. Predictors whose importance was significantly higher than the maximum importance of shadow variables were classified as significantly important. For readability, labels are shown only for important predictors and shadow variables.

Random forest analyses with Boruta feature selection confirmed the results of regression analyses. In a random forest model using 118 microbiological predictors along with the 2 major clinical predictors identified by regularized logistic regression (Table 3), the first four predictors by decreasing importance were age, IDU, *sedjr* and CC30 (see Supplementary Table 3 for a ranking of all predictors). The Boruta feature selection algorithm classified the same 4 predictors as significantly important (Figure 3), while all other predictors including *mecA* were classified as either unimportant or of undetermined importance.

## Discussion

This combined analysis of clinical and microbiological characteristics in 98 patients with *S. aureus* IE identified two bacterial genetic factors, namely, the absence of plasmid-borne genes encoding enterotoxins D, J, and R and the CC30 genetic background, as major predictors of the risk of embolism independent of host-related factors.

The clinical predictors of embolism included in multivariate analysis, such as a younger age, IDU, Charlson comorbidity index or vegetation size, were comparable to those repeatedly identified in cohorts from several countries from 2003 to 2014 (Durante Mangoni et al., 2003; Fowler et al., 2005; Thuny et al., 2005, 2007; Rizzi et al., 2014). This suggests that our cohort, although investigated in 2008–2009, remained representative in terms of embolism risk factors. It is intriguing that vegetation size, which was previously reported as an independent risk factor of embolism (Thuny et al., 2005), was discarded from the multivariate model. Similarly, the superantigen-encoding *tst1* gene was not significantly associated with embolism in spite of an OR estimate of 6.4 in univariate analysis (Table 2). We suspect that the limited sample size (*n* = 98) did not afford sufficient statistical power, resulting in the detection of only the strongest effects. Thus, we do not rule out a potential role of *tst1*, vegetation size or other clinical predictors such as IDU in our setting.

Our observation that a CC30 genetic background was an independent risk factor of embolism (adjusted OR, 9.7) brings further clinical support to the hypothesis of a peculiar pathogenic potential of CC30 in hematogenous infections (Messina et al., 2016). CC30 was overrepresented in IE compared to skin infections in the US, Europe and Middle-East (Fowler et al., 2007; Nienaber et al., 2011) but not in Australia (Nethercott et al., 2013). CC30 prevalence was moderate (~10%) in French cohorts of IE patients including the present cohort (Tristan et al., 2012; Bouchiat et al., 2015), however it is unknown whether this prevalence differs from that of CC30 in other infections. In a recent study of IE patients from Spain, CC30 was associated with persistent bacteremia (≥5 days) and negatively associated with death (Fernández-Hidalgo et al., 2017). Although the mechanism underlying the association of CC30 with IE is unclear, it has been suggested that several characteristics of CC30 contribute to a lower immune response, allowing in turn CC30 isolates to reach the bloodstream and survive within it (Spaulding et al., 2012). These immune escape mechanisms include an overall lower toxic and pro-inflammatory potential compared to other CCs, due to hampered alpha-toxin production (McGavin et al., 2012) and the expression of a phenol-soluble modulin variant with attenuated toxic and chemotactic activities (Cheung et al., 2014). Interestingly, a lower toxic potential was shown to strongly enhance *S. aureus* fitness in bloodstream (Laabei et al., 2015). Whether and how the lower toxic potential of CC30 influences embolus release remains an open question.

Patients infected with strains harboring the plasmid-borne *sedjr* genes had a reduced risk of developing embolism, independent of clinical factors and other *S. aureus* characteristics. The protective effect size was large (adjusted OR, 0.07), however the biological interpretation of *sedjr* involvement is not straightforward. Enterotoxins D, J, and R have been involved in food poisoning (Zhang et al., 1998). Interestingly, *sed* was previously found to belong to a group of enterotoxin genes (with *tst1, sea, see* and *sei*) overrepresented in IE isolates compared to soft tissue infection isolates in an international cohort (Nienaber et al., 2011), suggesting a possible involvement in IE development; however, embolism was not considered in the study. The superantigenic activity of *sedjr*-encoded toxins is unlikely to protect from embolism because other superantigens, such as TSST-1 or *egc*-harbored enterotoxins, have been shown to promote vegetation growth in animal models (Stach et al., 2016), which in turn favors embolism (Thuny et al., 2005). Moreover, other superantigen-encoding markers were widely distributed in our collection (Supplementary Table 2) but not associated with embolism. Collectively, these observations indicate that the negative association of *sedjr* with embolism is either due to a specific and yet-unknown function of enterotoxins D, J, or R, or to a spurious association with a causative gene or allele co-localized with *sedjr* on the same pIB485-like plasmid (Shearer et al., 2011). We favor the second interpretation for two reasons. First, *sedjr*-positive isolates belonged to two different CCs (Figure 1), suggesting that genetic features common to these isolates were plasmid-borne rather than chromosomic. Second, the *sedjr*-harboring pIB485-like plasmids contain numerous additional genes of unknown function (Shearer et al., 2011), including a *marR* family transcriptional regulator protein that could play a role in virulence by regulating chromosome-encoded genes (Alekshun and Levy, 1999; McCallum et al., 2004). Future research, possibly using isogenic variants harboring the pIB485-like plasmid in an experimental endocarditis model, should seek to determine whether plasmid-borne determinants influence the risk of IE-related embolism.

To conclude, genomic features of *S. aureus*, namely a CC30 genetic background and the absence of the *sedjr*-harboring plasmid present in some CC5 and CC8 strains, predicted embolism in *S. aureus* endocarditis. We also hypothesize that the *mecA* determinant of methicillin resistance, previously reported to be negatively associated with embolism (Hill et al., 2008), could have been so due to its association with *sedjr* markers. Collectively, our results suggest that the influence of *S. aureus* genotypic features on the risk of embolism may be stronger than previously suspected and independent of clinical risk factors. Determining the biological underpinnings of how *S. aureus* lineage- and plasmid-specific genes influence embolism would help to develop prognostic tools and preventive strategies.

## AEPEI study group on infective endocarditis

AEPEI: Association pour l'Etude et la Prévention de l'Endocardite Infectieuse – French Association for the Study and Prevention of Infective Endocarditis.

**Principal investigators**: B. Hoen and X. Duval. **Other members**: F. Alla, A. Bouvet, S. Briancxon, E. Cambau, M. Celard, C. Chirouze, N. Danchin, T. Doco-Lecompte, F. Delahaye, J. Etienne, B. Iung, V. Le Moing, J. F. Obadia, C. Leport, C. Poyart, M. Revest, C. Selton-Suty, C. Strady, P. Tattevin, and F. Vandenesch. **Coordinating investigators in the study regions**: Y. Bernard, S. Chocron, C. Chirouze, B. Hoen, P. Plesiat, I. Abouliatim, C. De Place, P. Tattevin, M. Revest, P. Y. Donnio, F. Alla, J. P. Carteaux, T. Doco-Lecompte, C. Lion, N. Aissa, C. Selton-Suty, B. Baehrel, R. Jaussaud, P. Nazeyrollas, C. Strady, V. Vernet, E. Cambau, X. Duval, B. Iung, P. Nataf, C. Chidiac, M. Celard, F. Delahaye, J. F. Obadia, F. Vandenesch, H. Aumaître, J. M. Frappier, V. Le Moing, E. Oziol, A. Sotto, and C. Sportouch. **Centre National de Référence des Streptocoques**: C. Poyart and A. Bouvet. **Centre National de Référence des Staphylocoques**: F. Vandenesch. M. Celard, and M. Bes. **Investigators**: P. Abassade, E. Abrial, C. Acar, N. Aissa, J. F. Alexandra, N. Amireche, D. Amrein, P. Andre, M. Appriou, M. A. Arnould, P. Assayag, A. Atoui, F. Aziza, N. Baille, N. Bajolle, P. Battistella, S. Baumard, A. Ben Ali, J. Bertrand, S. Bialek, M. Bois Grosse, M. Boixados, F. Borlot, A. Bouchachi, O. Bouche, S. Bouchemal, J. L. Bourdon, A. Bouvet, L. Brasme, F. Bricaire, E. Brochet, J. F. Bruntz, A. Cady, J. Cailhol, M. P. Caplan, B. Carette, J. P. Carteaux, O. Cartry, C. Cazorla, M. Celard, H. Chamagne, H. Champagne, G. Chanques, J. Chastre, B. Chevalier, C. Chirouze, F. Chometon, C. Christophe, A. Cohen, N. Colin de Verdiere, N. Danchin, V. Daneluzzi, L. David, P. De Lentdecker, F. Delahaye, V. Delcey, P. Deleuze, E. Donal, X. Duval, B. Deroure, V. Descotes-Genon, K. Didier Petit, A. Dinh, V. Doat, F. Duchene, F. Duhoux, M. Dupont, S. Ederhy, O. Epaulard, M. Evest, J. F. Faucher, B. Fantin, E. Fauveau, T. Ferry, M. Fillod, T. Floch, T. Fraisse, J. M. Frapier, L. Freysz, B. Fumery, B. Gachot, S. Gallien, I. Gandjbach, P. Garcon, A. Gaubert, J. L. Genoud, S. Ghiglione, C. Godreuil, A. Grentzinger, L. Groben, D. Gherissi, P. Gue'ret, A. Hagege, N. Hammoudi, F. Heliot, P. Henry, S. Herson, B. Hoen, P. Houriez, L. Hustache-Mathieu, O. Huttin, S. Imbert, B. Iung, S. Jaureguiberry, M. Kaaki, A. Konate, J. M. Kuhn, S. Kural Menasche, A. Lafitte, B. Lafon, F. Lanternier, V. Le Chenault, V. Le Moing, C. Lechiche, S. Lefèvre-Thibaut, A. Lefort, A. Leguerrier, J. Lemoine, L. Lepage, C. Leport, C. Lepouse', J. Leroy, P. Lesprit, L. Letranchant, D. Loisance, G. Loncar, C. Lorentz, P. Mabo, I. Magnin-Poull, T. May, A. Makinson, H. Man, M. Mansouri, O. Marcxon, J. P. Maroni, V. Masse, F. Maurier, M. C. Meyohas, P. L. Michel, C. Michelet, F. Mechaï, O. Merceron, D. Messika-Zeitoun, Z. Metref, V. Meyssonnier, C. Mezher, S. Micheli, M. Monsigny, S. Mouly, B. Mourvillier, O. Nallet, P. Nataf, P. Nazeyrollas, V. Noel, J. F. Obadia, E. Oziol, T. Papo, B. Payet, A. Pelletier, P. Perez, J. S. Petit, F. Philippart, E. Piet, C. Plainvert, B. Popovic, J. M. Porte, P. Pradier, R. Ramadan, M. Revest, J. Richemond, M. Rodermann, M. Roncato, I. Roigt, O. Ruyer, M. Saada, J. Schwartz, C. Selton-Suty, M. Simon, B. Simorre, S. Skalli, F. Spatz, C. Strady, J. Sudrial, L. Tartiere, A. Terrier De La Chaise, M. C. Thiercelin, D. Thomas, M. Thomas, L. Toko, F. Tournoux, A. Tristan, J. L. Trouillet, L. Tual, A. Vahanian, F. Verdier, V. Vernet Garnier, V. Vidal, P. Weyne, M. Wolff, A. Wynckel, N. Zannad, and P. Y. Zinzius.

## Author contributions

FV, FA, and CLep conceived and designed the study. AL and MB performed the experiments. AT, MB, CLec, and VV-G collected the data. J-PR, FA, and CLa analyzed the data. J-PR and AL drafted the manuscript. J-PR, FA, FV, and CLep take responsibility for the integrity of data analysis.

### Conflict of interest statement

The authors declare that the research was conducted in the absence of any commercial or financial relationships that could be construed as a potential conflict of interest.
