# Supplementary material for: Staphylococcus aureus CC30 Lineage and Absence of sed,j,r-Harboring Plasmid Predict Embolism in Infective Endocarditis
Source: Front Cell Infect Microbiol. 2018 Jun 8;8:187. doi: 10.3389/fcimb.2018.00187 (PMC6003251; doi:10.3389/fcimb.2018.00187)
Supplement: Supplementary file 1 [file Table_1.PDF]

Supplementary Table 1. Characteristics of 132 patients with *Staphylococcus aureus* endocarditis and comparison between the 98 patients included and the 34 patients excluded according to availability of *Staphylococcus aureus* strain.

	Whole population (n = 132) n (%)[a]	Patients with a genetic analysis of *S. aureus* (*n = 98*) n (%)[a]	NA	Patients without a genetic analysis of *S. aureus* (n = 34) n (%)[a]	NA	P value[b]
**Clinical and biological manifestations of IE**						
Fever	127 (96.2)	94 (95.9)	0	33 (97.1)	0	1.00
Location of IE (not exclusive)						
Aortic	47 (35.6)	35 (35.7)	0	12 (35.3)	0	1.00
Mitral	57 (43.2)	38 (38.8)	0	19 (55.9)	0	0.11
Tricuspid	28 (21.2)	23 (23.5)	0	5 (14.7)	0	0.34
Pacemaker	6 (4.5)	4 (4.1)	0	2 (5.9)	0	0.65
Unknown	9 (6.8)	7 (7.1)	0	2 (5.9)	0	1.00
Severe regurgitation	39 (30.2)	26 (27.1)	2	13 (39.4)	1	0.20
Heart failure	41 (31.1)	31 (31.6)	0	10 (29.4)	0	1.00
Septic shock (before surgery)	16 (12.1)	11 (11.2)	0	5 (14.7)	0	0.56
CRP at admission, mg/L, median (IQR)	226.5 (126-316.5)	228 (133-316)	8	197 (99-317)	4	0.47[c]
Immunologic phenomena	13 (10.2)	7 (7.4)	4	6 (18.2)	1	0.10
Creatinin serum levels ≥180 µmol/L	60 (46.2)	42 (43.8)	2	18 (52.9)	0	0.43
**Echocardiography**						
Vegetation (echocardiography)	118 (89.4)	87 (88.8)	0	31 (91.2)	0	1.00
Initial size of the vegetation, mm, median (IQR)	13 (10-20)	14 (10-20)	23	10 (8-16.5)	10	0.06[c]
Ordinal size of the vegetation, 5 classes						
No vegetation	14 (10.6)	11 (11.2)		3 (8.8)		
< 10 mm	24 (18.2)	15 (15.3)		9 (26.5)		
≥ 10 mm - < 15 mm	30 (22.7)	23 (23.5)	0	7 (20.6)	0	0.59[d]
≥ 15 mm	48 (36.4)	38 (38.8)		10 (29.4)		
unknown size	16 (12.1)	11 (11.2)		5 (14.7)		
Prosthesis dehiscence	3 (13.6)	1 (7.1)	84	2 (25.0)	26	0.53
Cardiac abscess	21 (15.9)	13 (13.3)	0	8 (23.5)	0	0.18
**Outcome**						
Cardiac surgery	48 (36.4)	34 (34.7)	0	14 (41.2)	0	0.54
Length of hospitalization, days, median (IQR)	39 (25-62.5)	37.5 (24-67)	0	42 (29-62)	0	0.29[c]
In-hospital death	53 (40.2)	42 (42.9)	0	11 (32.4)	0	0.32
Death at 1 year	61 (46.2)	48 (49.0)	0	13 (38.2)	0	0.32
**Embolic events**						
Embolic event (at least one)	71 (53.8)	54 (55.1)	0	17 (50.0)	0	0.69
Cerebral embolism	35 (26.5)	25 (25.5)	0	10 (29.4)	0	0.66
Symptomatic	30 (22.7)	22 (22.4)	0	8 (23.5)	0	1.00
Pulmonary embolism	24 (18.2)	20 (20.4)	0	4 (11.8)	0	0.31
Symptomatic	15 (11.5)	13 (13.4)	1	2 (5.9)	0	0.35
Other locations	39 (29.5)	29 (29.6)	0	10 (29.4)	0	1.00
Symptomatic	16 (12.6)	10 (10.6)	4	6 (18.2)	1	0.36

CRP, C-reactive protein; HD, heart disease; ICD, implantable cardiovertor defibrillator; IDU, injection drug use; IE, infective endocarditis; IQR, interquartile range; NA, numbers of patients with none available data; PM, pacemaker; *S. aureus*, *Staphylococcus aureus*.

[a]Values are numbers (percentages) unless otherwise indicated.

[b]P value from Fisher exact test, except [c]from Kruskal Wallis and [d]from Chi-squared.
