# Supplementary material for: Staphylococcus aureus CC30 Lineage and Absence of sed,j,r-Harboring Plasmid Predict Embolism in Infective Endocarditis
Source: Front Cell Infect Microbiol. 2018 Jun 8;8:187. doi: 10.3389/fcimb.2018.00187 (PMC6003251; doi:10.3389/fcimb.2018.00187)
Supplement: Supplementary file 2 [file Table_2.PDF]

Supplementary Table 2. Virulence determinants and genetic background of 98 *S. aureus* infective endocarditis isolates.

	Description	No. of isolates
**Virulence regulation**		
agr I	accessory gene regulator allele I	50 (51.0)
agr II	accessory gene regulator allele II	33 (33.7)
agr III	accessory gene regulator allele III	14 (14.3)
agr IV	accessory gene regulator allele IV	1 (1.0)
**Superantigens**		
tst 1	toxic shock syndrome toxin 1	9 (9.2)
sea	enterotoxin A (=entA)	23 (23.5)
seb	enterotoxin B (=entB)	1 (1.0)
sec	enterotoxin C (=entC)	15 (15.3)
sed	enterotoxin D (=entD)	13 (13.3)
seg	enterotoxin G (=entG)	54 (55.1)
seh	enterotoxin H (=entH)	6 (6.1)
sei	enterotoxin I (=entI)	53 (54.1)
sej	enterotoxin J (=entJ)	13 (13.3)
sek	enterotoxin K (=entK)	2 (2.0)
sel	enterotoxin L (=entL)	14 (14.3)
selm	enterotoxin-likegene/protein M (=sem, entM)	54 (55.1)
seln	enterotoxin-likegene/protein N (=sen, entN)	54 (55.1)
seo	enterotoxin-like gene/protein O (=seo, entO)	54 (55.1)
seq	enterotoxin Q (=entQ)	2 (2.0)
ser	enterotoxin R (=entR)	13 (13.3)
selu	enterotoxin-likegene/protein U (=seu, entU)	55 (56.1)
**Cytotoxins**		
luk S	haemolysin gamma / leukocidin, component C (S)	81 (82.7)
luk D	leukocidin D component	62 (63.3)
luk E	leukocidin E component	56 (57.1)
luk X	leukocidin/ haemolysin toxin family protein	93 (94.9)
luk Y	leukocidin/haemolysin toxin family protein	72 (73.5)
**Multilocus sequence typing (MLST) assignment**		
CC5		19 (19.4)
CC45		15 (15.3)
CC8		15 (15.3)
CC15		11 (11.2)
CC30		11 (11.2)
CC398		5 (5.1)
CC7		4 (4.1)
ST6		4 (4.1)
CC1		2 (2.0)
CC20		2 (2.0)
CC25		2 (2.0)
CC97		2 (2.0)
Others[a]		6 (6.1)

CC, clonal complex.

[a]Including CC10, CC12, CC121, CC88, CC9 and ST188, n=1 each.
