# Supplementary material for: Staphylococcus aureus CC30 Lineage and Absence of sed,j,r-Harboring Plasmid Predict Embolism in Infective Endocarditis
Source: Front Cell Infect Microbiol. 2018 Jun 8;8:187. doi: 10.3389/fcimb.2018.00187 (PMC6003251; doi:10.3389/fcimb.2018.00187)
Supplement: Supplementary file 3 [file Table_3.PDF]

Supplementary Table 3. Ranking of predictors of embolism by decreasing importance in random forest model.

Predictor	Importance
Age	46.41
Intravenous drug use	42.82
seDJR	31.28
CC30	22.76
mecA	20.64
mecAdelta_mecRugpQcum	20.36
delta_mecR	20.32
agrIII__total_	19.43
mecAdelta_mecRugpQ	19.06
ugpQ	18.10
agr_class	15.62
qacA	15.34
hla	15.18
agr	15.17
CC20	14.94
cnasdrD	14.21
ccrA_2	13.80
Q9XB68_dcs	13.65
lukE	13.58
bbp__total_	12.93
aadD	12.81
blaZ	12.70
lukS	12.57
lukS__ST22_ST45_	12.12
ccrB_2	11.27
msrA	10.13
sasG__total_	10.06
CC97	9.69
ccrB_4	8.79
CC8ST72	8.18
mapsdrDcum	8.13
entGIMNotherOUcum	7.78
agrII__total_	7.42
agrI__total_	7.06
ermC	6.91
chp	6.05
cnasdrDcum	5.92
lukY__ST30_ST45_	5.91
cna	5.21
cap_8___total_	4.59
sdrD__total_	4.13
cap_5__total_	4.04
CC45	3.88
ccrA_1	3.51
splB	3.42
fnbB__total_	3.41
lukY_	3.27

ccrA_4	3.00
entO	2.92
aur__MRSA252_	2.87
entN__cons_	2.51
aur__Other_than_MRSA252_	2.32
CC8	2.28
Q6GD50__putat__fusidic_acid_resi	2.25
lukD	1.63
splE	1.58
entI	0.79
entGIMNconsOU	0.34
CC5	0.29
entGIMNotherOU	0.13
agrIV__total_	0.00
ccrB_1	0.00
kdpA_SCC	0.00
kdpB_SCC	0.00
kdpC_SCC	0.00
kdpD_SCC	0.00
kdpE_SCC	0.00
mpbBM	0.00
vatA	0.00
vgb	0.00
aphA3	0.00
sat	0.00
far1	0.00
cat___total_	0.00
entB	0.00
arcA_SCC	0.00
arcB_SCC	0.00
arcC_SCC	0.00
arcD_SCC	0.00
bap	0.00
CC10	0.00
CC12	0.00
CC121	0.00
CC88	0.00
CC9	0.00
ST188	0.00
mapsdrD	0.00
entL	-0.02
entN__other_than_RF122_	-0.05
splA	-0.08
entG	-0.41
entM	-0.42
entU	-0.44
tst1___human__allele_	-1.14
entC	-1.50
entA	-1.84
tst1__consensus_	-2.06

aur__cons_	-2.50
CC15	-3.33
scn	-3.84
entCM14_probe2	-3.99
ST6	-4.04
map__total_	-4.05
qacC___total_	-4.57
entGIMNconsOUcum	-4.85
entCM14_probe1	-5.40
tetK	-5.90
CC7	-6.45
sak	-6.64
ermA	-7.18
CC25	-7.48
entK	-8.08
lukX	-8.44
entQ	-9.47
edinB	-9.50
etD	-9.53
cc1	-9.61
entA__N315____entP	-12.02
CC398	-12.13
entH	-15.42
